# Intra-articular synovial giant cell tumor of the hip: a rare case report

**DOI:** 10.1093/jscr/rjaf102

**Published:** 2025-02-28

**Authors:** Mario Cahueque, Carlos R Arriaza

**Affiliations:** Department of Orthopedics and Traumatology, Hospital Centro Médico, 6A Avenida 3-47, Guatemala City, Guatemala; Department of Orthopedics and Traumatology, Hospital Centro Médico, 6A Avenida 3-47, Guatemala City, Guatemala

**Keywords:** synovial giant cell tumor, hip, tumor, arthroscopy

## Abstract

Synovial giant cell tumor (SGCT) is a rare, locally aggressive neoplasm that primarily affects joints, bursae, and tendon sheaths. While commonly associated with conditions involving bone, its occurrence within the hip joint is exceedingly rare. Here, we present a case of a 34-year-old female with an intra-articular SGCT of the hip, detailing the surgical intervention, histopathological findings, and postoperative outcomes.

## Introduction

Synovial giant cell tumor (SGCT) is categorized into two forms: localized (involving tendon sheaths) and diffuse (affecting synovial membranes). The localized variant, commonly referred to as giant cell tumor of the tendon sheath, is well-documented, whereas the diffuse form primarily affects synovial membranes and joints. This condition often presents with chronic joint pain, swelling, and restricted mobility. Although the etiology remains unclear, it is believed to involve inflammatory and neoplastic processes, with recurrent mutations in genes such as CSF1 and COL6A3 implicated in the pathogenesis [1, 2].

## Case presentation

A 34-year-old female presented with a 6-month history of progressive left groin pain. Initially, the pain was intermittent but progressively worsened, impacting her ability to perform daily activities. Physical examination revealed severe tenderness in the left hip with restricted abduction and flexion. Both the FABER (Flexion, Abduction, External Rotation) and FADIR (Flexion, Adduction, Internal Rotation) tests were nonassessable due to intolerable pain.

**Figure 1:**
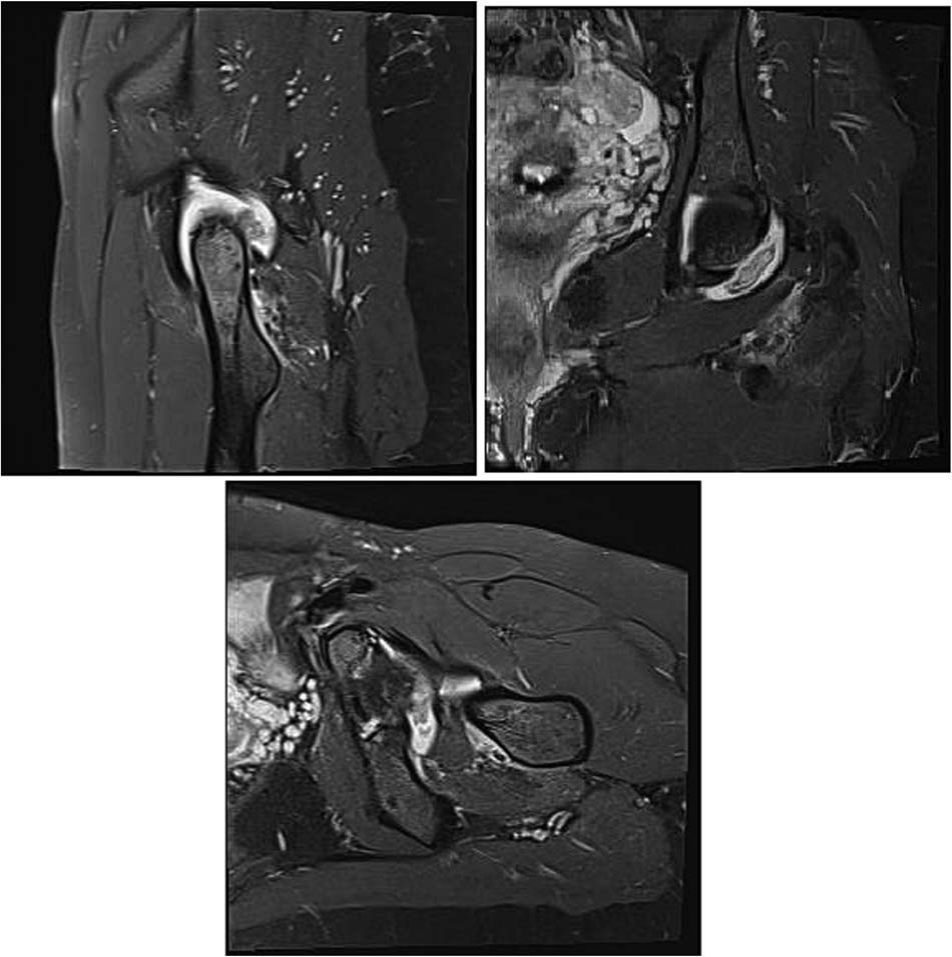
MRI is observed in axial, sagittal, and coronal sections, showing the presence of an intra-articular lesion and abundant synovitis.

Magnetic resonance imaging (MRI) of the hip revealed a well-defined intra-articular mass with extensive synovial thickening (Fig. 1). Given these findings, the patient underwent hip arthroscopy to remove the mass and perform a histopathological examination.

### Surgical intervention

**Figure 2:**
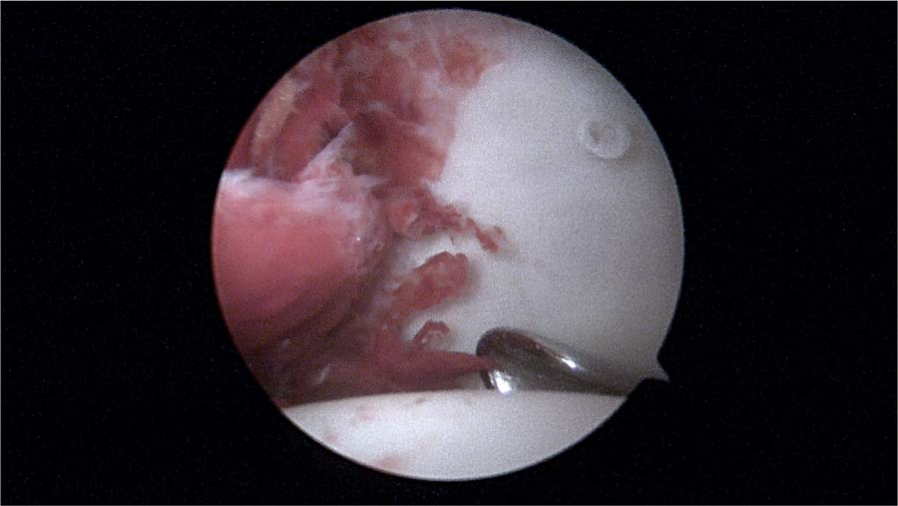
Arthroscopy image showing a large intra-articular lesion.

**Figure 3:**
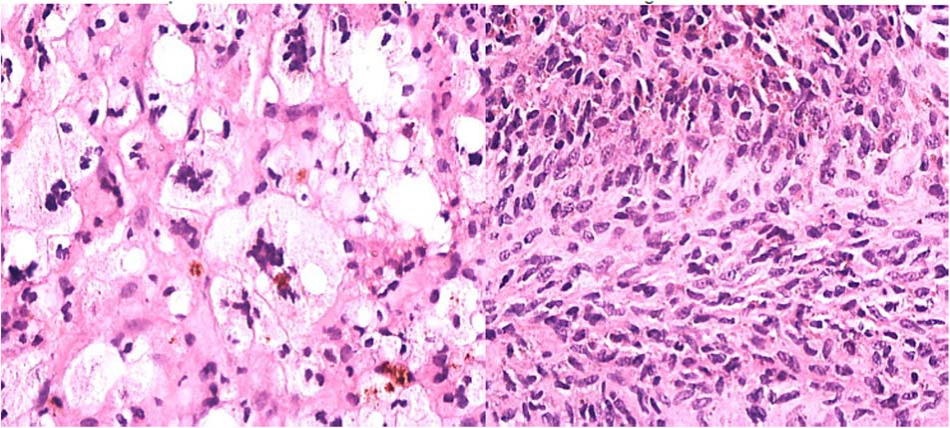
Histopathology showing proliferation of mononuclear synovial cells with oval or polygonal shapes and multinucleated giant cells resembling osteoclasts.

During the arthroscopic procedure, extensive synovial proliferation and a well-defined mass were identified within the joint space. The mass was excised, and the synovium was sampled for histopathological evaluation (Fig. 2). The pathology report confirmed the diagnosis of an SGCT, a rare entity within the hip joint (Fig. 3).

### Histopathological findings

The histopathological examination revealed a nodular proliferation of mononuclear and multinucleated giant cells, consistent with an SGCT. These tumors typically exhibit mild to moderate cellular atypia without significant mitotic activity, which contributes to their localized and aggressive nature. The tumor’s rare occurrence in synovial tissue underscores its complexity and the need for precise diagnostic methods [3].

### Outcome and follow-up

Postoperatively, the patient reported significant pain relief and restored hip mobility. At the 1-month follow-up, her symptoms had improved substantially, allowing her to resume daily activities without discomfort. One year after surgery, the patient remains asymptomatic with no clinical or radiological evidence of recurrence. However, given the nature of SGCT, there is a risk of recurrence. In such cases, total hip replacement may be considered, especially if recurrence occurs or if conservative management fails [4].

## Discussion

SGCTs are rare, locally aggressive benign neoplasms primarily affecting joints, bursae, and tendon sheaths. While typically associated with tendon sheaths, their presentation within the hip joint is exceedingly rare. In this case, we describe the successful management of an intra-articular SGCT through hip arthroscopy, with a thorough review of the literature to contextualize this case.

The diagnosis and management of SGCTs in the hip pose unique challenges due to their rarity and the limited number of cases reported. Typically, SGCTs present with chronic joint pain, swelling, and restricted mobility, similar to other synovial disorders. However, distinguishing SGCTs from other conditions such as labral tears, synovitis, or osteoarthritis is critical, as misdiagnosis can lead to inappropriate treatment strategies.

Several studies have contributed to our understanding of SGCTs. Rao and Vigorita [1] emphasized that although benign, SGCTs have a locally aggressive behavior, necessitating complete surgical excision to minimize recurrence risk. Furthermore, the recurrence rates have been reported to range between 8% and 46% in various studies, underscoring the need for long-term monitoring [2, 3].

Arthroscopic management of SGCTs has been increasingly utilized due to its minimally invasive nature and diagnostic accuracy. This approach allows for precise removal of the tumor while preserving joint function. In comparison, open surgical techniques may involve greater risks of complications and joint stiffness, which can impair postoperative recovery. Studies have shown that arthroscopic intervention provides better preservation of joint function and a lower risk of recurrence [4].

Our case highlights that despite successful tumor excision and symptom relief, continuous monitoring is essential. The risk of recurrence, as reported in the literature, mandates regular clinical and imaging follow-ups. In this regard, advanced imaging techniques such as MRI are pivotal for early detection of recurrence, allowing timely intervention [5].

Comparing this case with other similar reports, it becomes evident that while outcomes for SGCTs in the hip are generally positive, the potential for recurrence remains a significant concern. Bertoni *et al*. [6] found that patients with intra-articular SGCTs in other joints experienced higher rates of recurrence compared with those undergoing complete excision, further supporting the role of thorough surgical management.

In conclusion, this case underscores the importance of arthroscopic intervention for SGCTs in the hip, emphasizing the role of histopathological confirmation and long-term follow-up to manage potential recurrences effectively. As more cases are reported, the need for standardized treatment approaches will become even more apparent.

## Conclusion

This case underscores the importance of recognizing SGCTs within the hip joint, a rare yet clinically significant condition. Through arthroscopic techniques, effective treatment and diagnosis can be achieved, offering significant relief to patients. However, the potential for recurrence necessitates ongoing surveillance and, in certain cases, advanced intervention such as total hip replacement.
